# Gap-plasmon based broadband absorbers for enhanced hot-electron and photocurrent generation

**DOI:** 10.1038/srep30650

**Published:** 2016-07-29

**Authors:** Yuhua Lu, Wen Dong, Zhuo Chen, Anders Pors, Zhenlin Wang, Sergey I. Bozhevolnyi

**Affiliations:** 1 College of Physics, Optoelectronics and Energy, Collaborative Innovation Center of Suzhou Nano Science and Jiangsu Key Laboratory of Thin Films, Soochow University, Suzhou 215006, China; 2 School of Physics and National Laboratory of Solid State Microstructures, Nanjing University, Nanjing 210093, China; 3 Centre for Nano Optics, University of Southern Denmark, Campusvej 55, DK-5230 Odense M, Denmark

## Abstract

Plasmonic hot-electron generation has recently come into focus as a new scheme for solar energy conversion. So far, however, due to the relatively narrow bandwidth of the surface plasmon resonances and the insufficient resonant light absorption, most of plasmonic photocatalysts show narrow-band spectral responsivities and small solar energy conversion efficiencies. Here we experimentally demonstrate that a three-layered nanostructure, consisting of a monolayer gold-nanoparticles and a gold film separated by a TiO_2_ gap layer (Au-NPs/TiO_2_/Au-film), is capable of near-completely absorbing light within the whole visible region. We show that the Au-NPs/TiO_2_/Au-film device can take advantage of such strong and broadband light absorption to enhance the generation of hot electrons and thus the photocurrent under visible irradiation. As compared to conventional plasmonic photocatalysts such as Au-NPs/TiO2 nanostructures, a 5-fold-enhanced incident photon-to-current conversion efficiency is achieved within the entire wavelength range 450–850 nm in the Au-NPs/TiO_2_/Au-film device. Simulations show good agreements with the experimental results, demonstrating that only the plasmon-induced losses contribute to the enhanced photocurrent generation of the Au-NPs/TiO_2_/Au-film device.

Semiconductor-based solar photocatalysis for sustainable and clean chemical fuel production has attracted a considerable interest in the past decades due to its great potential for resolving energy and environmental issues^1^. Since the pioneering work of Fujishima and Honda in the 1970s^2^, *n*-type titania (TiO_2_) has shown superior performance in terms of photocatalytic ability, chemical stability, earth abundance and cost effectiveness, becoming one of the most commonly used semiconductor photoelectrodes^1^,^2^,^3^,^4^,^5^,^6^,^7^. The main drawback of TiO_2_ is its wide-bandgap (~3.2 eV for anatase), which limits its photo-absorption to the ultraviolet (UV) range of the solar spectrum and, consequently, suppresses its overall photocatalytic efficiency. Considerable efforts have been applied to expand the energy utilization of TiO_2_ to the visible region, including substitutional element doping^8^,^9^,^10^, defect creation^11^,^12^, organic dye sensitization^13^,^14^,^15^, and heterojunction formation^16^,^17^,^18^, the latter representing a direct technical route as opposed to the development of narrow-bandgap semiconductors having comparable performance with TiO_2_ in all other aspects^19^.

Recently, plasmonic (i.e., involving surface plasmons) energy conversion has come into focus as a new scheme for solar energy conversion^20^,^21^. In the plasmonic photocatalysts composed of metal nanostructures and semiconductors, highly energetic or hot electrons, generated from the non-radiative decay of surface plasmon resonances (SPRs), can escape from the metal nanostructures before their thermalization and be injected via a Schottky barrier, formed at the metal-semiconductor interface, into the conduction band of the semiconductor^21^. One interesting property of this process is that the photon energy should only be larger than the Schottky barrier, rather than the conventional semiconductor bandgap^22^. Although various metal-TiO_2_ nanostructures have been demonstrated to exhibit visible-light response by exploiting the hot-electron injection mechanism^20^,^21^,^23^,^24^,^25^,^26^,^27^,^28^,^29^,^30^, they featured narrow-band spectral responsivities and, consequently, limited solar energy conversion efficiencies, due to insufficient light absorption via narrow-band SPRs. Plasmonic systems using thin metal film coated *n*-type Si substrates with deep trench cavities or square block patterns were shown to exhibit strong broadband optical absorption in the near-infrared region, resulting in a large enhancement in the photoresponsivity well below the semiconductor band edge^31^,^32^. However, the implementation of these structures relies on high-cost and time-consuming lithography approaches that are also not suitable for large-area fabrication.

Very efficient light absorption in the visible range was demonstrated by using three-layered metal-dielectric-metal geometries with a lithographically patterned top metal layer^33^,^34^,^35^. The physical origin of this absorption lies in the fact that the metal-dielectric-metal nanostructures are capable of supporting gap plasmon resonances and, consequently, inducing equivalent electric and effective magnetic surface currents, which produce reflected waves that interfere destructively^36^. This mechanism makes the obtained near-complete absorption insensitive to the angle and polarization of incidence, and particularly insensitive to the spatial order or periodicity^36^. Furthermore, it has been shown that such near-complete absorption could acquire a broadband nature by exploiting differently-sized metal nanoparticles (NPs)^37^. The aforementioned remarkable features indicate that the broadband near-perfect absorption can be achieved in the metal-dielectric-metal structures with randomly distributed and differently sized metal NPs, whose fabrication is feasible on mass-production scale using non-lithographic ways^38^,^39^,^40^.

In this study, by integrating the metal-dielectric-metal based broadband near-perfect visible-light absorber with a wide-bandgap semiconductor TiO_2_, we experimentally demonstrate a stable plasmonic photocatalyst with largely enhanced generation of hot electrons arising from plasmon decay. Our implementation is based on the standard sputtering and thermal annealing techniques, thus keeping fabrication simple and cost-effective. Although both the intrinsic losses and the plasmon-induced losses contribute to the total absorption, we demonstrate that only the contribution from the plasmon-induced absorption can be taken advantage of to enhance the photocurrent generation. We also show that within the entire wavelength range 450–850 nm, the measured incident photon-to-current conversion efficiency (IPCE) of the Au-NPs/TiO_2_/Au-film device is 5-fold higher than that of the conventional plasmonic structure consisting of a TiO_2_ film decorated with gold NPs (Au-NPs/TiO_2_).

## Results and Discussion

**Figure 1:**
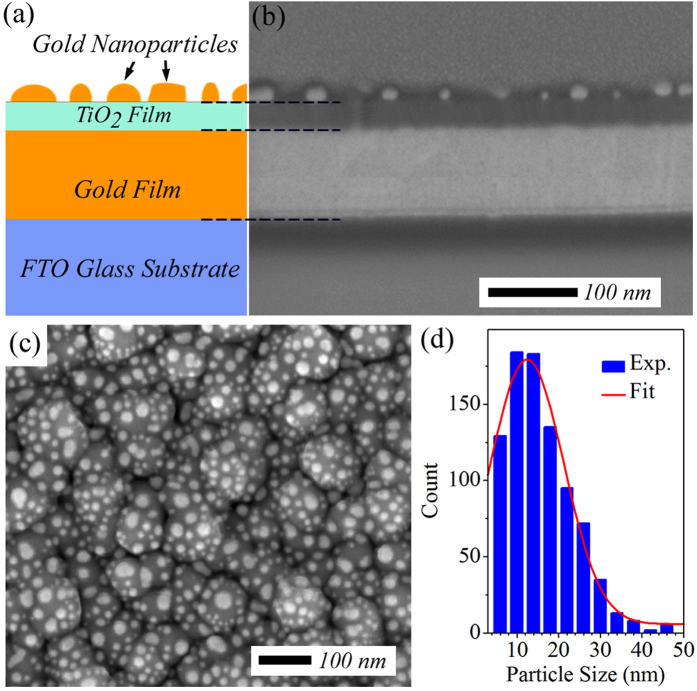
(**a**) Schematic of the metal-dielectric-metal nanostructure that is composed of (from bottom to the top) a FTO glass substrate coated with an optically thick Au film (~150 nm) followed by a thin TiO_2_ film acting as the spacer layer and at the very top, a monolayer of Au NPs with random size dispersion and spatial distribution. (**b**) Side-view SEM image of the optimized Au-NPs/TiO_2_/Au-film nanostructure prepared with a 5-nm-thick Au film pre-deposited on a 50-nm-thick TiO_2_ spacer layer. (**c**) Top-view SEM image of the optimized Au-NPs/TiO_2_/Au-film nanostructure. (**d**) Histogram of effective size of the Au NPs formed after the thermal treatment.

As schematically shown in Fig. 1a, the near-perfect visible absorber employed in this study is a three-layered plasmonic nanostructure supported by a fluorine-doped tin oxide (FTO) glass substrate. The bottommost layer of the structure is an optically thick Au film with a thickness of ~150 nm, and the middle layer is a thin TiO_2_ semiconductor film. At the very top, there is a monolayer of Au NPs formed by thermal annealing of a very thin Au film pre-deposited on the TiO_2_ layer, in which the surface coverage and size dispersion of the NPs can be controlled by the initial Au film thickness and the thermal annealing condition^41^. Previous studies have already demonstrated that the gap layer thickness and the surface coverage and size dispersion of metal NPs could affect the overall absorption of the metal-dielectric-metal nanostructures^36^,^37^,^38^,^39^. Considering that the thermal annealing process is also intentionally used here to transform the as-grown amorphous TiO_2_ film to polycrystalline anatase film structure (see Fig. S1 in Supporting Information), we perform the thermal treatment in air atmosphere at 400 °C for 3 hours. Under this fixed annealing condition, the absorption is therefore determined by the thicknesses of the TiO_2_ gap layer and the pre-deposited Au film. The optimum values of these two thicknesses leading to the strongest visible light absorption with the broadest possible bandwidth can be easily found through multiple deposition experiments (see Methods). Figure 1b,c show respectively the side-view and top-view scanning electron microscope (SEM) images of the optimized Au-NPs/TiO_2_/Au-film nanostructure prepared with a 5-nm-thick Au film pre-deposited on a 50-nm-thick TiO_2_ spacer layer. These SEM images reveal that the Au NPs with large size dispersion are distributed randomly on the TiO_2_ layer after the thermal annealing. Further analysis on the top-view SEM image (Fig. 1c) shows that the Au NPs occupy ~35% of the surface area and their sizes follow a Gaussian distribution centered at 12 nm (Fig. 1d).

**Figure 2:**
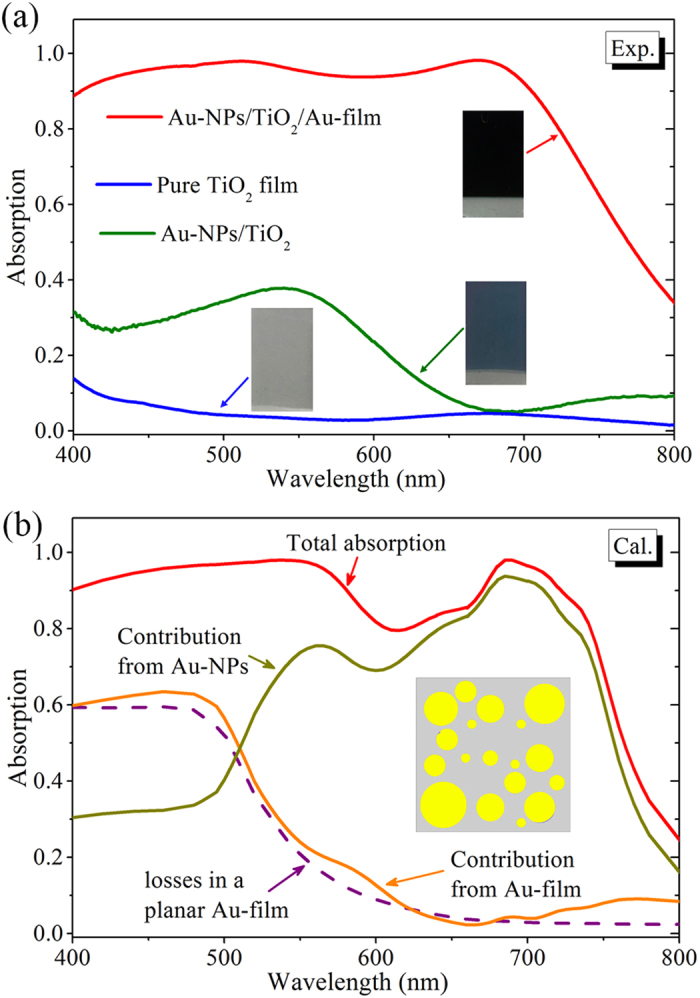
(**a**) Experimentally measured absorption spectra of the Au-NPs/TiO2/Au-film nanostructure (red), the pure TiO_2_ film (blue), and the Au-NPs/TiO_2_ nanostructure (olive). The insets show the photographic images of the prepared samples. (**b**) Calculated normal incidence absorption spectra of the Au-NPs/TiO_2_/Au-film nanostructure, and absorption contributions from the top layer of Au NPs (dark-yellow) and the bottom Au film (orange). For comparison, simulated absorption spectrum of a planar optically thick Au film is also present (purple dashed line). The inset shows the unit cell with a period of 100 nm and surface coverage of ~35%.

To characterize the optical properties of the fabricated samples, the reflection and transmission are measured in an optical microscope coupled to a visible/near-infrared spectrometer with absorption calculated as *A* = 1–Transmission–Reflection (see Methods for detailed optical measurements). Figure 2a displays the absorption spectra of the optimized Au-NPs/TiO_2_/Au-film nanostructure. For direct comparison, Fig. 2a also shows the absorption spectra of two control samples: a pure 50-nm-thick TiO_2_ film and a Au-NPs/TiO_2_ nanostructure. As expected, the pure TiO_2_ film only shows negligible absorption in the visible range (blue curve in Fig. 2a). For the Au-NPs/TiO_2_ nanostructure, optical absorption with a maximum value of 40% (around the wavelength of 550 nm) and a full-width half-maximum (FWHM) of ~200 nm is observed (olive curve in Fig. 2a), which is due to the excitations of the localized-SPRs supported by Au NPs^20^,^21^,^23^,^24^,^25^,^26^,^27^,^28^,^29^,^30^. As compared to the Au-NPs/TiO_2_ nanostructure, the optimized Au-NPs/TiO_2_/Au-film nanostructure appears quite dark in colour (insets of Fig. 2a), and exhibits remarkably high ~90% average absorption in the entire visible spectrum 400–750 nm. Furthermore, the optical absorption of the optimized Au-NPs/TiO_2_/Au-film nanostructure exhibits a negligible incident angle dependency (see Fig. S2 in Supporting Information), which is consistent with previously reported studies^36^,^37^,^38^,^39^.

Figure 2b shows the calculated normal incidence absorption spectrum of the Au-NPs/TiO_2_/Au-film nanostructure, which is conducted by using the three-dimensional finite-element-method (FEM) software COMSOL Multiphysics. In the simulations, the Au-NPs/TiO_2_/Au-film nanostructure is approximated by a periodic super cell configuration consisting of a monolayer of circularly-shaped 20-nm-thick Au disks with diameters randomly chosen from the analyzed Gaussian distribution (Fig. 1d) and a semi-infinite Au film separated by a 50-nm-thick TiO_2_ planar film. The subwavelength super cell period of 100 nm is determined from the measured surface coverage of ~35% (inset of Fig. 2b). The dielectric constant of TiO_2_ is set to 3.9, and the permittivity of Au is taken from the experimental data of Johnson and Christy^42^. It should be noted that the simulated absorption, especially within the wavelength range 600–700 nm, is slightly weaker than the measured absorption, which may result from the approximations made in the simulations, such as smoothed TiO_2_ gap layer, Au NPs that are assumed to have circular shapes and uniform heights, and the imparted periodicity that is not present in the fabricated structure. Apart from this discrepancy, the calculated and measured results are in reasonable agreement with each other.

Previous studies have demonstrated that both the intrinsic losses and the plasmon-induced losses, which should respectively reside in the bottom metal layer and the top layer of metal NPs, could contribute to the absorption obtained from the metal-dielectric-metal nanostructures^37^,^43^. However, these two loss mechanisms could play different roles in the generation of hot electrons, as will be demonstrated later. Therefore, it is necessary to separately quantify contributions from these two different mechanisms in the Au-NPs/TiO_2_/Au-film nanostructure. To do this, the absorption contributions from the top layer of Au NPs and the bottom Au film are calculated by integrating the power loss density over their respective volumes and shown in Fig. 2b. The absorption contribution from the bottom Au film (orange curve in Fig. 2b) is found to coincide with the absorption spectrum calculated for an optically thick planar Au film (purple curve in Fig. 2b), implying that the intrinsic losses are only affected weakly by the gap-plasmon resonances in the Au-NPs/TiO_2_/Au-film nanostructure. Furthermore, it is also seen that the intrinsic loss is larger than the absorption contribution from the top layer of Au NPs within the wavelength range 400–517 nm. On the contrary, for the wavelengths longer than ~517 nm, the absorption contribution from the top layer of Au NPs becomes exceedingly larger than the absorption contribution from the bottom Au film, revealing that the plasmon-induced absorption is the dominant loss mechanism in this wavelength range.

**Figure 3:**
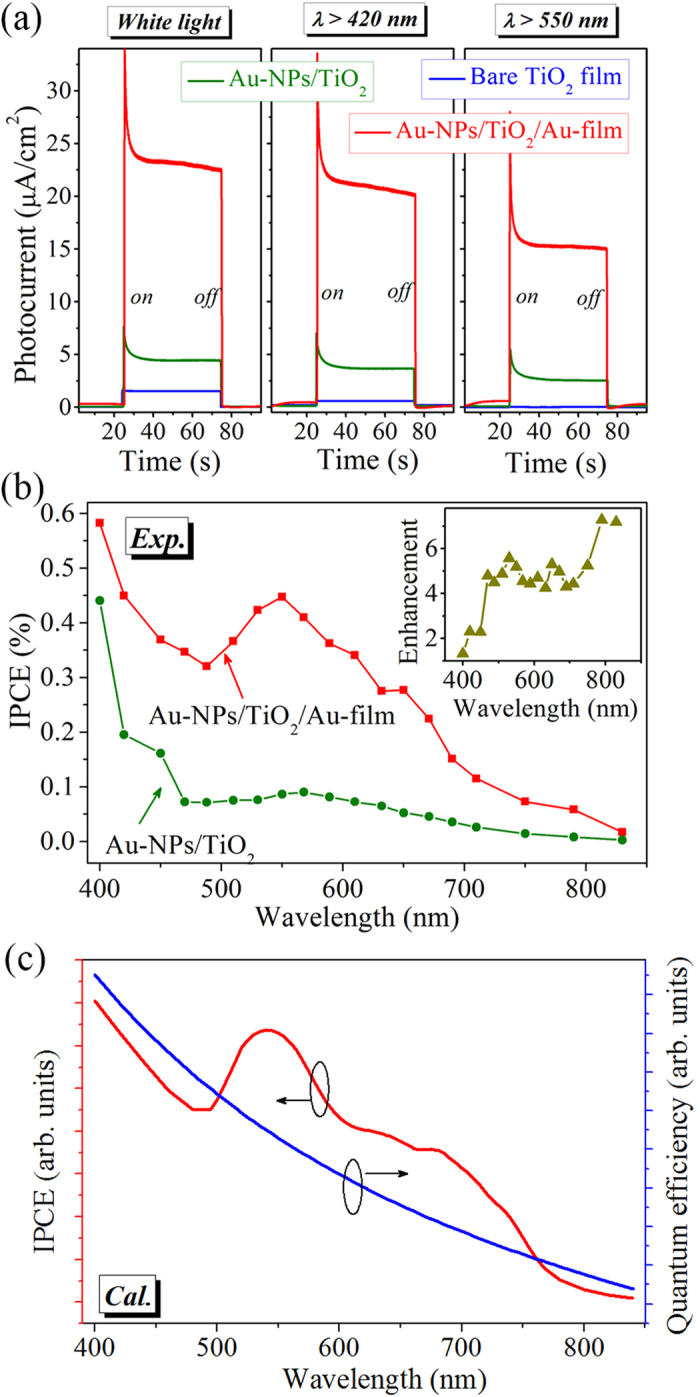
(**a**) Photocurrent under zero external bias voltage versus time plots for the Au-NPs/TiO_2_/Au-film nanostructure (red), the bare TiO_2_ film (blue), and the Au-NPs/TiO_2_ (olive) illuminated by the full solar spectrum and by visible light. (**b**) Experimentally measured IPCE spectra of the Au-NPs/TiO_2_/Au-film nanostructure (red) and the Au-NPs/TiO_2_ (olive). The inset shows the IPCE enhancement ratio of the Au-NPs/TiO_2_/Au-film nanostructure to the Au-NPs/TiO_2_. (**c**) Quantum efficiency of hot electrons across the Au-TiO_2_ Schottky barrier (blue) and simulated IPCE spectrum of the Au-NPs/TiO_2_/Au-film nanostructure (red).

To evaluate the PEC performance of the prepared samples, the short-circuit photocurrent measurements are carried out in a three-electrode system under zero external bias voltage (see Methods for detailed photocurrent measurements). Figure 3a shows the photocurrent versus time (*I*-*t*) curves for the Au-NPs/TiO_2_/Au-film nanostructure (red curve), the pure TiO_2_ film (blue curve), and the Au-NPs/TiO_2_ nanostructure (olive curve) under illuminations of white light and visible light (*λ* > 420 nm and *λ* > 550 nm). The bare TiO_2_ film produces a photocurrent density of ~1.5 μA/cm^2^ under light illumination of full spectrum (including UV region), and an ultra-small photocurrent density of ~0.02 μA/cm^2^ under visible illumination (*λ* > 550 nm), which is consistent with the negligible visible light absorption of the TiO_2_ film (blue curve in Fig. 2a). However, due to the existence of possible vacancy defects or chemical impurities^30^, our prepared TiO_2_ film is found to generate a relatively small photocurrent density of ~0.58 μA/cm^2^ under visible illumination (*λ* > 420 nm). When the TiO_2_ film is decorated with Au NPs, corresponding to its enhanced visible light absorption, the observed photocurrent densities are increased to ~4.4 μA/cm^2^, ~3.7 μA/cm^2^ and ~2.6 μA/cm^2^ under light illuminations of full spectrum, *λ* > 420 nm and *λ* > 550 nm, respectively. Similar photocurrent enhancements have been observed in the previously reported metal-TiO_2_ nanostructures and have been attributed to the hot electrons generated from the non-radiative decay of the localized-SPRs in the Au-NPs^20^,^21^,^23^,^24^,^25^,^26^,^27^,^28^,^29^,^30^. With the goal of expanding the energy utilization of TiO_2_ to the visible light based on the hot electron injection mechanism, it is necessary to increase the number of hot electrons generated upon light absorption by the plasmonic nanostructures. As compared to the two control samples, the Au-NPs/TiO_2_/Au-film nanostructure possesses much stronger light absorption (Fig. 2a), and therefore, is expected to correspondingly exhibit higher plasmonic-induced visible-light photoresponsivity. It is seen from Fig. 3a that the Au-NPs/TiO_2_/Au-film nanostructure indeed exhibits the highest photocurrent density among all the three samples. Even under the visible (*λ* > 550 nm) illumination, the recorded photocurrent density in the Au-NPs/TiO_2_/Au-film nanostructure is as high as ~15.8 μA/cm^2^, which is consistent with the value obtained from the linear sweep voltammagram (see Fig. S3 in Supporting Information) and is ~6 times the one obtained from the Au-NPs/TiO_2_ nanostructure under otherwise the same conditions.

Figure 3b shows the experimental IPCE spectra of the Au-NPs/TiO_2_/Au-film nanostructure (red line) and the Au-NPs/TiO_2_ under zero external bias voltage. At each center wavelength (*λ*, unit in nm) of a quasi-monochromatic light produced using a bandpass filter (10-nm-FWHM), the IPCE value is defined as IPCE = 1240*R*/*λ*, where *R* = *I*/*J* is the photoresponsivity, with *I* being the measured photocurrent density (mA/cm^2^) and *J* being the incident light power density (mW/cm^2^). The Au-NPs/TiO_2_/Au-film device shows much higher IPCE values than the Au-NPs/TiO_2_, which coincides with the stronger broadband visible light absorption of the Au-NPs/TiO_2_/Au-film nanostructure. As shown in the inset of Fig. 3b, the IPCE enhancement ratio of the Au-NPs/TiO_2_/Au-film nanostructure to the Au-NPs/TiO_2_ more clearly demonstrates that the enhancement is significant and reaches a particular high factor of ~5 over the entire wavelength range 450–850 nm.

As already demonstrated above, both the intrinsic losses and the plasmon-induced losses contribute to the absorption of the Au-NPs/TiO_2_/Au-film nanostructure (Fig. 2b). For the metal-dielectric-metal architecture shown in Fig. 1a, Schottky junctions can be formed at the interfaces between the TiO_2_ film and the top layer of the Au NPs as well as the bottom Au film. These Schottky barriers allow hot electrons to be collected and injected from both the top and bottom Au-parts into the conduction band of TiO_2_, producing photocurrents with opposite flow directions^44^, provided that hot electrons can be generated by the above mentioned different loss mechanisms. At short wavelengths (*λ* < 517 nm), because the intrinsic losses in the bottom Au film are larger than the plasmon-induced losses in the top layer of Au NPs (Fig. 2b), more hot electrons are expected to be transmitted from the bottom contact to the top contact than in the opposite direction. By contrast, it is expected to see that hot electrons are mainly transmitted from the top to the bottom contact at long wavelengths (*λ* > 517 nm), owing to the dominant plasmon-induced losses in the top layer of Au NPs (Fig. 2b). However, the detected photocurrents under illumination of quasi-monochromatic light with wavelengths of 450 nm and 650 nm are found to have the same flow directions, running from the top to bottom contact (see Fig. S4 in Supporting Information). This implies that the intrinsic losses have almost no contribution to the generation of hot electrons (and thus the photocurrent). As a result, only the plasmon-induced losses in the top layer of Au NPs need to be considered in the hot electron generation and transfer process. According to the modified Fowler relation^22^, the quantum efficiency of hot electrons across the Schottky barrier can be approximated as *η* = *C*_*F*_(*hν* − *qϕ*_*b*_)^2^/*hν*, with *C*_*F*_ being the Fowler emission coefficient, *hυ* being the photon energy and *qϕ*_*b*_ being the barrier height. Figure 3c (blue line) shows the calculated quantum efficiency spectrum of the Au-TiO_2_ junction with a barrier height of *qϕ*_*b*_ ≈ 0.96 eV 19, which reveals much higher value in the shorter wavelengths and decreases gradually as increasing the incident wavelengths. With the already obtained spectrum of the absorption contribution from the top layer of Au NPs (*A*) and the above-calculated quantum efficiency spectrum (*η*), the photoresponsivity spectrum *R* can be given by *R* = *Aη* 22, and consequently the IPCE spectrum can be calculated as IPCE = 1240 *R*/*λ*. The line shape of the calculated IPCE spectrum of the Au-NPs/TiO_2_/Au-film nanostructure (red solid line in Fig. 3c) is very similar to the experimentally measured one (red line in Fig. 3b), clearly demonstrating that the gap-plasmon supported by the Au-NPs/TiO_2_/Au-film nanostructure plays a key role in the PEC performance improvement.

**Figure 4:**
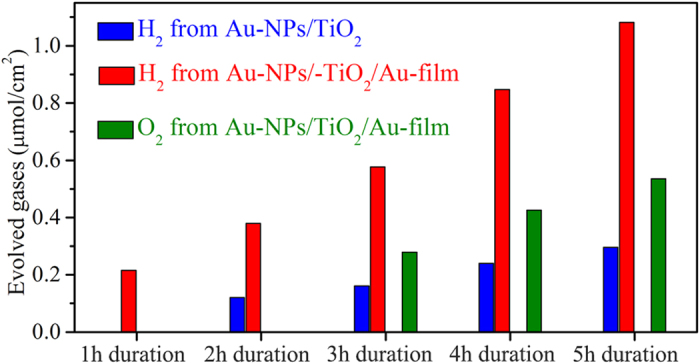
The evolved hydrogen (red) and oxygen (olive) from the Au-NPs/TiO_2_/Au-film nanostructure, and the evolved hydrogen (blue) from the Au-NPs/TiO_2_ nanostructure under visible-light illumination (*λ* > 550 nm) as a function of illumination duration.

The PEC water splitting performances of the prepared Au-NPs/TiO_2_/Au-film and Au-NPs/TiO_2_ nanostructures are also directly examined (see Methods for detailed hydrogen and oxygen measurements). Figure 4 shows both the hydrogen and oxygen evolution under illumination of visible light (*λ* > 550 nm) as a function of illumination duration. For the Au-NPs/TiO_2_ nanostructure, hydrogen production could not be detected after 1 h-illumination. When the illumination duration is increased to 2 h–5 h, the hydrogen generation density evolved from the Au-NPs/TiO_2_ nanostructure is found to be about 0.12, 0.16, 0.24, and 0.30 μmol/cm^2^, respectively (blue patterns in Fig. 4). By linearly fitting the dependence of the hydrogen generation density on the illumination duration, the hydrogen generation rate is found to be ~0.06 μmol/h∙cm^2^. It should be noted that even after 5 h visible light illumination the amount of the oxygen evolved from the Au-NPs/TiO_2_ nanostructure is still below the detection limit. For the Au-NPs/TiO_2_/Au-film device, the hydrogen generation density of ~0.22 μmol/cm^2^ is readily to be detected after 1 h illumination, and is found to be almost linearly increased from ~0.38 μmol/cm^2^ to ~1.08 μmol/cm^2^ with increasing the illumination duration from 2 h to 5 h (blue pattern in Fig. 4). The hydrogen generation rate obtained from linear fitting is ~0.21 μmol/h∙cm^2^ for the Au-NPs/TiO_2_/Au-film device, which is over 3 times the one obtained from the Au-NPs/TiO_2_ nanostructure. Furthermore, the oxygen generation density of ~0.28 μmol/cm^2^ is detected from the Au-NPs/TiO_2_/Au-film nanostructure after 3 h visible light illumination, and is increased from ~0.43 μmol/cm^2^ to ~0.54 μmol/cm^2^ with increasing the illumination duration from 4 h to 5 h (olive pattern in Fig. 4). The oxygen generation rate obtained from linear fitting is ~0.11 μmol/h∙cm^2^, which is almost half of the obtained hydrogen generation rate (~0.21 μmol/h∙cm^2^), and thus confirming that the hydrogen and oxygen are evolved at the expected stoichiometric ratio in PEC water-splitting experiment. The above results clearly demonstrate that the Au-NPs/TiO_2_/Au-film nanostructure exhibits much improved PEC performance, which in turn confirms that increasing the absorption of incident photons in the plasmonic catalysts can enhance the generation of hot electrons and photocurrents. It is also worth noting that no noticeable degradation in the PEC performance of the Au-NPs/TiO_2_/Au-film device is observed after 5 sequential 1 h–5 h runs (total 15 h-illumination), which is consistent with previously reported study^24^,^29^ and indicates that the operational lifetime of Au-TiO_2_ based plasmonic photocatalysts could exceed that of the efficient water splitters based on semiconductors^29^.

In summary, we experimentally demonstrate that the Au-NPs/TiO_2_/Au-film nanostructure is capable of providing a broadband near-perfect visible light absorption. Such greatly enhanced broadband light absorption in the Au-NPs/TiO_2_/Au-film nanostructure could overcome the shortcomings such as the narrowband and insufficient resonant light absorption in the conventional plasmonic-TiO_2_ photocatalysts^20^,^21^,^23^,^24^,^25^,^26^,^27^,^28^,^29^,^30^, and thus could be exploited to enhance the generation of hot electrons and photocurrents. We show that a 5-fold-enhanced incident photon-to-current conversion efficiency is achieved within the entire wavelength range 450–850 nm in the Au-NPs/TiO_2_/Au-film device, as compared to the Au-NPs/TiO_2_ nanostructures. Although the achieved efficiency of the plasmonic device is still lower than that of the conventional solar cells due to the loss of hot electrons via ultrafast electron-electron scattering^21^, our present work can give an impetus to the fabrication of simple nanostructures for hot-electron based plasmonic catalysts. For practical applications, the PEC performance of the plasmonic device needs to be improved, which may be accomplished by introducing some other enhancement mechanisms, such as oxygen evolution catalyst^26^, roughening the metal/semiconductor interface^45^, and using graphene as hot electron receiver^46^. In particular, plasmon induced interfacial charge transfer transition has been recently reported as a new hot-electron transfer mechanism with a quantum yield for electron injection of 24%^47^. We hope that combing this fast hot-electron injection mechanism into the metal-dielectric-metal based plasmonic photocatalysts with near-perfect broadband absorption will allow the efficiency of the plasmonic device in excess of 24%.

## Methods

Au and TiO_2_ films were deposited by radio frequency (RF) magnetron sputtering. Cleaned FTO glasses (1.5 × 2 cm^2^) were used as supporting substrates and placed parallel to the target at a distance of 60 mm. The chamber pressure was first pumped down to 1 × 10^−4^ Pa, then argon was introduced to a pressure of 1.5 Pa. The TiO_2_ and Au targets were powered by an RF generator at a frequency of 13.56 MHz, and were pre-sputtering in argon for 3 minutes before each deposition step. The control sample of the pure TiO_2_ film was prepared by depositing TiO_2_directly onto the FTO substrate. The control sample of the Au-nanoparticles loaded TiO_2_ film was prepared by successively depositing TiO_2_ and Au onto the FTO substrate. For preparing the Au-NPs/TiO_2_/Au-film nanostructures, an optically thick (150 nm) Au layer was first deposited onto the FTO substrate. After that, TiO_2_ and Au were successively sputtered onto the as-deposited Au layer. The thicknesses of the TiO_2_ layer and the top layer Au film were varied to find the optimum values to make the Au-NPs/TiO_2_/Au-film nanostructure achieve the strongest visible light absorption with the broadest possible bandwidth. All the as-prepared samples were annealed in air atmosphere at 400 °C for 3 hours to form the Au nanoparticles and simultaneously transform the amorphous TiO_2_ film to polycrystalline anatase film structure.

The reflection and transmission spectra measurements were performed with an optical microscope. The samples were illuminated by un-polarized white light from a halogen lamp focused by a microscope objective. The reflected light from the samples was collected by the same objective lens, while the transmitted light was collected by a microscope condenser. The collected light was then analyzed by a visible/near-infrared spectrometer. The measured transmission spectra (*T*) were normalized with a transmission spectrum of a cleaned FTO glass substrate, and the reflection spectra (*R*) were normalized with a reflection spectrum collected on a silver mirror. For the bare TiO_2_ film and the Au-nanoparticles loaded TiO_2_ film (two control samples), the experimental absorption spectra were derived by *A* = 1 − *T* − *R*. The bottom Au film in the Au-NPs/TiO_2_/Au-film nanostructure is thick enough to eliminate the optical light transmission (*T* = 0). Therefore, only the reflection spectra needed to be measured for the Au-NPs/TiO_2_/Au-film nanostructures, and the absorption spectra were given by *A* = 1 − *R*.

To measure the photocurrent, all the prepared samples were connected to the electrochemical workstation (CHI 660E) as the working electrode. A platinum wire and a saturated calomel electrode (SCE) were used as the counter and reference electrodes, respectively. An aqueous KOH (1Mol l^−1^, PH = 14) was used as the electrolyte solution. The area of the sample exposed to light was ~0.25 cm^2^. The photocurrent measurements under illumination of white light were carried out directly with a 300 W xenon lamp source. Long-pass filters with a cut-on wavelength of 420 nm and 550 nm were placed in the light path to simulate the visible illuminations of *λ* > 420 nm and *λ* > 550 nm, respectively. Action spectrum was obtained by a semi-monochromatic irradiation produced through a series of band pass filters with different center wavelengths (10 nm full widths at half-maximum). The wavelength dependent light intensity was measured by a spectroradiometer (Newport 1918-C). To quantitatively determine the amount of evolved H_2_ and O_2_ resulting from the reduction of water, the three-electrode system same as that used in the photocurrent measurement was employed, in which an aqueous KOH solution (1Mol l^−1^, PH = 14) was also used as the electrolyte solution and the area of the sample exposed to light was ~2 cm^2^. The gas collected from the platinum counter electrode was analyzed by gas chromatography-mass spectroscopy (GC-MS).

## Additional Information

**How to cite this article**: Lu, Y. *et al.* Gap-plasmon based broadband absorbers for enhanced hot-electron and photocurrent generation. *Sci. Rep.* **6**, 30650; doi: 10.1038/srep30650 (2016).

## Supplementary Material

Supplementary Information
