# Supplementary material for: Gap-plasmon based broadband absorbers for enhanced hot-electron and photocurrent generation
Source: Sci Rep. 2016 Jul 29;6:30650. doi: 10.1038/srep30650 (PMC4965776; doi:10.1038/srep30650)
Supplement: Supplementary Information [file srep30650-s1.doc]

Supporting Information for

**Gap-plasmon based broadband absorbers for enhanced hot-electron and photocurrent generation**

Yuhua Lu ^1^, Wen Dong^1,*^, Zhuo Chen^2,*^, Anders Pors^3^, Zhenlin Wang^2^, and Sergey I. Bozhevolnyi^3,*^

^1^College of Physics, Optoelectronics and Energy, Collaborative Innovation Center of Suzhou Nano Science and Jiangsu Key Laboratory of Thin Films, Soochow University, Suzhou 215006, China

^2^School of Physics and National Laboratory of Solid State Microstructures, Nanjing University, Nanjing 210093, China

^3^Centre for Nano Optics, University of Southern Denmark, Campusvej 55, DK-5230 Odense M, Denmark

Correspondence and requests for materials should be addressed to W. D., Z.C. and S.I.B. (emails: dongwen@suda.edu.cn; zchen@nju.edu.cn; seib@iti.sdu.dk)

**Keywords:** hot electrons, plasmonic water splitting, broadband near-perfect absorption, gap surface plasmons


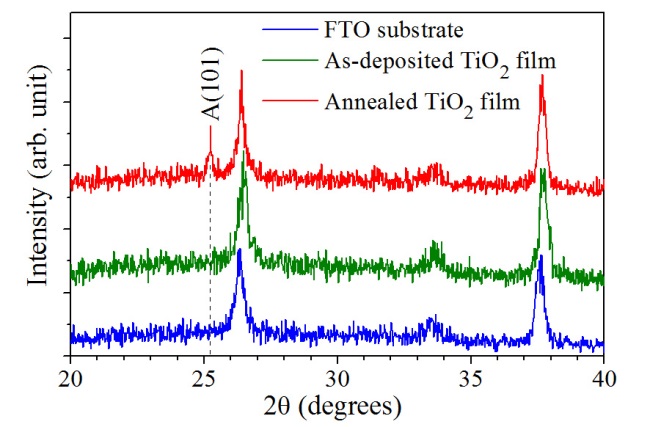


**Figure S1.** XRD patterns of the FTO glass substrate, the as-deposited TiO_2_ film and the annealed TiO_2_ film. The TiO_2_ film has a thickness of ~200 nm and is supported by the FTO glass. It can be seen that the XRD patterns of the as-deposited TiO_2_ film are the same as that of the bare FTO glass substrate, revealing the amorphous nature of the as-deposited TiO_2_ film. After annealing in air atmosphere at 400 °C for 3 hours, the TiO_2_ film shows an additional peak at 25.3°, which is corresponding to (101) index of anatase phase and therefore confirms that the TiO_2_ film has transformed from amorphousto polycrystalline anatase after the thermal treatment.


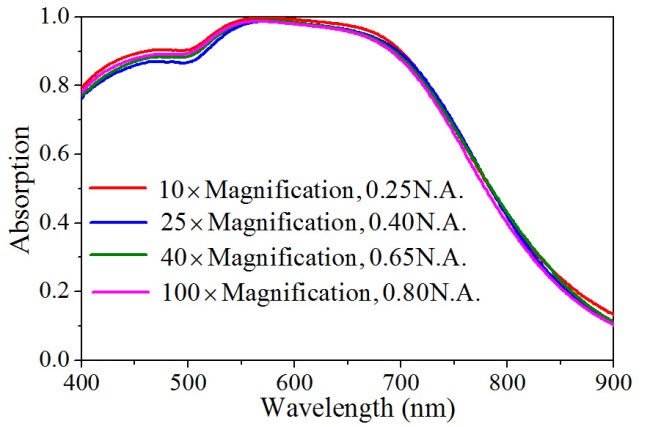


**Figure S2.** The absorption spectra of the Au-NPs/TiO_2_/Au-film nanostructure measured using different microscope objectives with different numerical apertures. Negligible (less than 4%) differences in light absorption were observed with these objective lenses, indicating that the optical absorption of the Au-NPs/TiO_2_/Au-film nanostructure is independent of the angle of incidence.


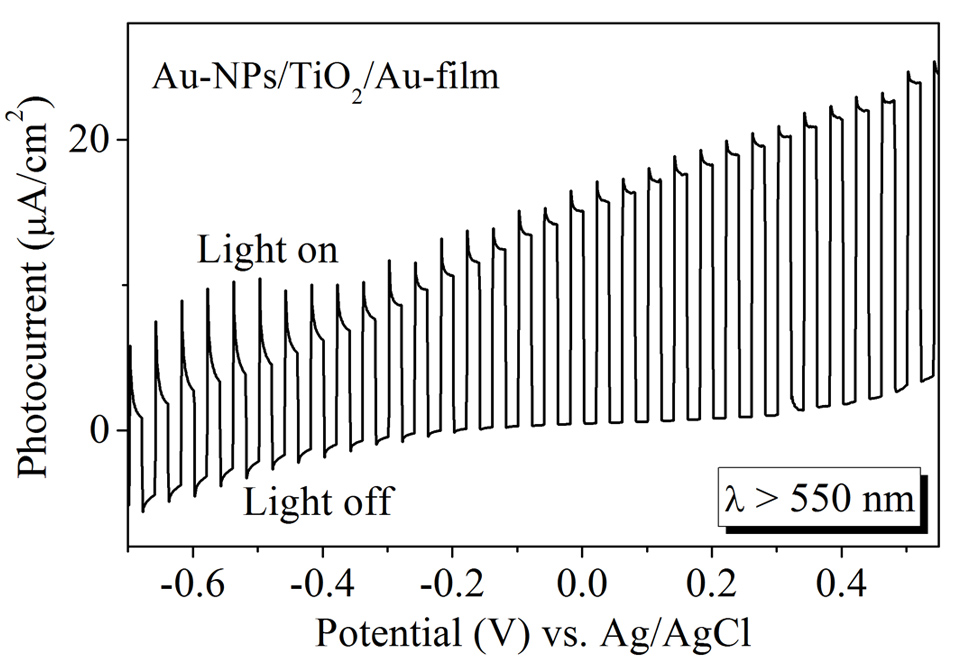


**Figure S3.** The measured I-V curve under chopped white light illumination, which allows the dark and light currents to be monitored simultaneously. In the measurement, the Au-NPs/TiO_2_/Au-film nanostructure is immersed in a solution of 1 M KOH (pH = 14), a Ag/AgCl electrode and a Pt wire are used as the reference and the counter electrode, respectively. The scan rate is 20 mV/s.


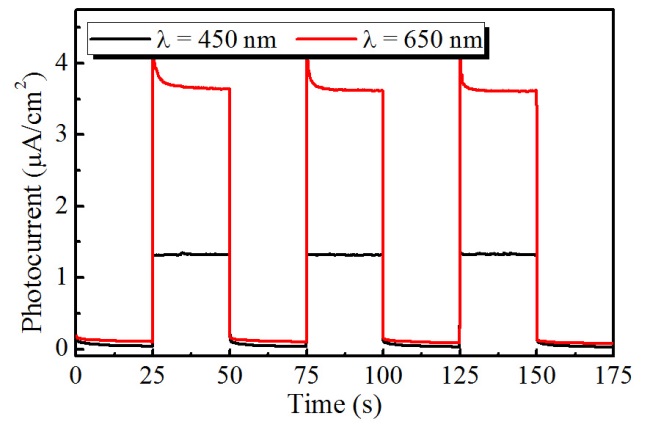


**Figure S4**. The photocurrents of the Au-NPs/TiO_2_/Au-film nanostructure measured under illuminations of quasi-monochromatic light with wavelengths of 450 nm and 650 nm. In both cases, the Au-NPs/TiO_2_/Au-film nanostructure only generates the anodic photocurrent that runs from the top to bottom contact.
